# Comparison of diagnostic tests for the detection of *Brucella *spp. in camel sera

**DOI:** 10.1186/1756-0500-4-525

**Published:** 2011-12-06

**Authors:** Mayada M Gwida, Adel H El-Gohary, Falk Melzer, Herbert Tomaso, Uwe Rösler, Ulrich Wernery, Renate Wernery, Mandy C Elschner, Iahtasham Khan, Meike Eickhoff, Daniel Schöner, Heinrich Neubauer

**Affiliations:** 1 Friedrich-Loeffler-Institut, Institute for Bacterial Infections and Zoonoses, Jena, Germany; 2 Department of Hygiene and Zoonoses, Faculty of Veterinary Medicine, Mansoura University, Mansoura, Egypt; 3 Freie Universität Berlin, Institute of Animal and Environmental Hygiene, Berlin, Germany; 4 Central Veterinary Research Laboratory, Dubai, United Arab Emirates; 5 Roche Diagnostics AG, Rotkreuz, Switzerland

## Abstract

**Background:**

Brucellosis in livestock causes enormous losses for economies of developing countries and poses a severe health risk to consumers of dairy products. Little information is known especially on camel brucellosis and its impact on human health. For surveillance and control of the disease, sensitive and reliable detection methods are needed. Although serological tests are the mainstay of diagnosis in camel brucellosis, these tests have been directly transposed from cattle without adequate validation. To date, little information on application of real-time PCR for detection of *Brucella *in camel serum is available. Therefore, this study was performed to compare the diagnostic efficiency of different serological tests and real-time PCR in order to identify the most sensitive, rapid and simple combination of tests for detecting *Brucella *infection in camels.

**Findings:**

A total of 895 serum samples collected from apparently healthy Sudanese camels was investigated. Sudan is a well documented endemic region for brucellosis with cases in humans, ruminants, and camels. Rose Bengal Test (RBT), Complement Fixation Test (CFT), Slow Agglutination Test (SAT), Competitive Enzyme Linked Immunosorbant Assay (cELISA) and Fluorescence Polarization Assay (FPA) as well as real-time PCR were used. Our findings revealed that *bcsp31 *kDa real-time PCR detected *Brucella *DNA in 84.8% (759/895) of the examined samples, of which 15.5% (118/759) were serologically negative. Our results show no relevant difference in sensitivity between the different serological tests. FPA detected the highest number of positive cases (79.3%) followed by CFT (71.4%), RBT (70.7%), SAT (70.6%) and cELISA (68.8%). A combination of real-time PCR with one of the used serological tests identified brucellosis in more than 99% of the infected animals. 59.7% of the examined samples were positive in all serological tests and real-time PCR. A subpopulation of 6.8% of animals was positive in all serological tests but negative in real-time PCR assays. The high percentage of positive cases in this study does not necessarily reflect the seroprevalence of the disease in the country but might be caused by the fact that the camels were imported from brucellosis infected herds of Sudan, accidentally. Seroprevalence of brucellosis in camels should be examined in confirmatory studies to evaluate the importance of brucellosis in this animal species.

**Conclusion:**

We suggest combining *bcsp31 *real-time PCR with either FPA, CFT, RBT or SAT to screen camels for brucellosis.

## Introduction

Camels are the most robust animal species in production and survival under harsh environmental conditions. Although many pastoral groups and communities throughout the world depend on camels for their livelihood, the health status of camels has not yet received proper attention from researchers and scientists. Brucellosis is caused by Gram-negative bacteria of the genus *Brucella *in man and animals. *Brucella *taxonomy and species discrimination rely on biochemical, antigenic, and metabolic characteristics. Camels are highly susceptible to *B. melitensis *and *B. abortus *[1] but camels are not known to be primary hosts of *Brucella*. *B. melitensis *bv 1-3 is predominantly isolated from sheep and goats, and *B. abortus *biovar 1-7 and 9 from cattle and other Bovidae. Thus, the infection of camel herds depends on the *Brucella *species prevalent in other animal species sharing the same habitats, and on husbandry methods [2]. The infection seems to be widespread among camel herds in Africa and on the Arabian Peninsula [3]. A comprehensive review on the seroprevalence in camels has recently been published by this working group. The clinical signs of brucellosis in camels are not clearly defined. Many infected camels are silent carriers of brucellosis. Consumption of *Brucella *infected food e.g. milk and meat from camels has led to a high number of human brucellosis cases and serious public health problems. Farmers from nomadic areas believe that raw camel milk has a curative effect on the digestive system [4].

Classical tests for the diagnosis of brucellosis i.e. culture and phenotypic characterization, are laborious, time-consuming, pose the risk of infection, and can generate discordant results. Isolation of the causing agent often fails in routine diagnosis. Serological tests are therefore commonly used for *Brucella *diagnosis in cattle and small ruminants especially at herd level, but cross-reactions with other Gram-negative bacteria are a major problem. Rose Bengal Test (RBT), Complement Fixation Test (CFT), and Slow Agglutination Test (SAT) are widely used for the detection of antibodies to *Brucella *spp. The sensitivity of RBT fulfills the requirements for surveillance of free areas at flock level but it is believed that only the combination of RBT and CFT in infected flocks can obtain accurate individual sensitivity in test-and-slaughter programs [5]. CFT is also recommended by World Organisation for Animal Health (OIE) as a test prescribed for international trade [6]. The CFT is recognized as a good test when correctly performed, but it has many practical drawbacks: it is cumbersome, time consuming and difficult to standardize [7]. None of the above mentioned tests can distinguish between antibodies produced after vaccination and those due to infection [8]. Different enzyme-linked immunosorbent assays (ELISA) have been developed to overcome these problems. Additionally, ELISA could detect *Brucella *carriers which were seronegative by RBT, SAT and CFT [9].

The fluorescence polarisation assay (FPA) is a recently described test used for the serological diagnosis of *Brucella *infection. It is a rapid, homogenous, species-independent assay, which was initially developed and validated for the detection of antibodies to *B. abortus *in cattle. FPA has many methodological advantages over the older, more established tests. It has yet to become established within the routine testing procedures of most National Brucellosis Reference Laboratories [10]. The FPA requires minimal manipulations and can be completed in few minutes [11]. It is assumed that serological tests used for *Brucella *infection in cattle may also be adequate for diagnosis of brucellosis in camels. However, no validation for camel sera was done yet.

Nucleic acid amplification methods might circumvent the diagnostic window being presented before production of specific antibodies. Real-time PCR offers improved sensitivity, specificity and speed of performance when compared to conventional PCR.

The present study was made to compare different serological tests and real-time PCR to define a rapid and simple technique capable of specifically detecting *Brucella *infection in camels.

## Materials and methods

### Samples

A total of 895 serum samples were received from Central Veterinary Research Laboratory (CVRL), Dubai, UAE. These samples were collected from apparently healthy camels *(Camelus dromedaries) *which were imported from Sudan at the end of 2008 and the beginning of 2009.

### Serological tests

All camel serum samples were tested by RBT, SAT, CFT, cELISA and FPA. Antigens used for RBT, SAT, and CFT were supplied by Institute Pourquier, France. Positive and negative control sera are the national reference sera standardized according to OIE. Positive control sera contain 421 I.U. per milliliter for SAT and 595 International CFT Units (ICFTU) per milliliter for CFT. RBT was conducted as described in the Manual of Standards for Diagnostic Tests and Vaccines [12] using antigen obtained from Institute Pourquier, France. SAT was performed in microtiter plates [13]. Samples showing more than 30 I.U. per milliliter were considered positive. The reagents used in the CFT were standardized and the test was performed according to OIE [12]. Any serum showing a value ≥ 20 ICFTU per milliliter was considered positive. The cELISA was done and results were interpreted according to the instructions of the manufactures using Svanovir™ *Brucella*-Ab cELISA kit (Svanovia Biotech AB Uppsala, Sweden). FPA was done and results were interpreted according to the instructions of the manufacturer (Diachemix, Whitefish Bay, WT, USA).

Briefly, the test protocols used in our study are the same as used for bovine brucellosis. Also serum dilution for FPA was chosen according to the manufacturer's protocol for bovine brucellosis. The positive control serum was of bovine origin.

### DNA preparation

DNA was purified using the High pure PCR Template preparation Kit ™ (Roche Diagnostics, Mannheim, Germany) according to the instructions of the manufacturer. 200 μl of serum were used in the assay. Subsequently, the concentration of DNA was determined photometrically using a Nano Drop ND-1000 UV-Vis spectrophotometer (Nano-Drop Technologies, Wilmington, DE, USA).

### Real-Time PCR

Real-time PCR for the genus specific *Brucella *cell surface salt extractable *bcsp31 *kDa protein gene was performed on DNA extracted from camel serum samples using the following primers (5'GCTCGGTTGCCAATATCAATGC 3') as forward primer and (5'GGGTAAAGCGTCGCCAGAAG 3') as reverse primer together with genus specific probe (5'6FAM-ACTCCAGAGCGCCCGACTTGATCG-DB 3') [14]. The primers and probes were obtained from TIB MOLBIOL (Berlin, Germany). The real-time PCR assay was prepared using the TaqMan™ Universal Master Mix (Applied Biosystems, New Jersey USA) containing the following components per reaction: 12.5 μl TaqMan™ Universal Master Mix (Applied Biosystems), 0.75 μl of each primer (0.3 μM) and 0.25 μl probe (0.1 μM). 2 μl of bacterial DNA was used as target and nuclease-free water sum up to a total reaction volume of 25 μl. No Template Controls (NTC) that contained 2 μl of water instead of DNA and positive controls that contained DNA of *Brucella *were included in each run to detect any amplicon contamination or amplification failure. The real-time PCR reaction was performed in duplicate in optical 96-well microtitre plates (q PCR 96-well plates, Micro Amp TM, Applied Biosystem) using a Mx3000P thermocycler system (Stratagene, La Jolla, Canada) with the following run condition 1 cycle of 50°C for 2 min, 1 cycle of 95°C for 10 min, followed by 50 cycles of 95°C for 25 s and 57°C for 1 min. The extracted DNA from the bcsp31 positive samples were examined with the *Brucella IS711 *species specific real-time PCRs for *B. abortus *and *B. melitensis *using the primers and probe as described previously [14] for typing. Amplification reaction mixtures were prepared in volumes of 25 μl containing 12.5 μl TaqMan™ Universal Master Mix (Applied Biosystems) 0.75 μl of each primer (0.3 μM) and 0.5 μl TaqMan probe (0.2 μM), 5 μl of template, and nuclease-free water sum up to a total reaction volume of 25 μl. Optimisation resulted in reaction condition of 2 min at 50°C, 10 min at 95°C, followed by 50 cycles of 95°C for 15 s and 57°C for 1 min. Cycle threshold values below 40 cycles were interpreted as positive. The threshold was set automatically by the instrument. The samples scored positive by the instrument were additionally confirmed by visual inspection of the graphical plots showing cycle numbers versus fluorescence values.

### Analytic Sensitivity

To determine the linear measuring range, five replicates of six 10 fold serial dilutions of *B. abortus *DNA in negative camel sera were assessed simultaneously in a single run. To determine the limit of detection, a probit analysis was performed using SPSS for Windows (Version 8.01, SPSS Inc., Chicago, USA). The probit analysis was applied to determine the number of genome equivalents that can be detected with a probability of 95%. Therefore, probit analysis was performed with the result of 8 replicates of the following amount of DNA per reaction: 1 pg, 100 fg, 50 fg, 20 fg, 10 fg, 5 fg and 1 fg, carried out on three different days. Based on the full bacterial genome sequence of *B. melitensis *published in GenBank (accession numbers NC_003317 and NC_003318), it was determined that each *Brucella *genome copy (i.e., genome equivalent [GE]) amounts to 3.38 fg according to the following equation: GE (fg) = number of base pairs per GE × 618 g mol^-1 ^× 10^15^/6.023 × 10^23 ^mol^-1 ^(Avogadro constant). The data were also used to determine repeatability and reproducibility of the assay.

### Statistical analysis

Data analyses were carried out using a statistical software program (SPSS for Windows, Version 17.01, SPSS Inc., Chicago, USA). The agreement between different serological tests was calculated using Kappa analysis.

As no gold standard was available we performed Latent Class Analysis (LCA) with all test results. The latent class model could not be applied for two subpopulations with different prevalences because samples from only one camel population were available, i.e. the camels imported from Sudan to UAE in 2008. However, we constructed a latent class model based on the status of "*Brucella *infection" (infected versus not infected animals) which can be regarded as a pseudo-gold standard or as presumed true status of infection with two latent classes. Using contingency tables and percentage agreements we determined total, positive, and negative correlation among test results and the latent class. The correlation was assessed using the Chi-Square test. Latent class analysis was conducted with R version 2.13 using the e1071 library.

## Results

FPA showed the highest number of positive samples 710 (79.3%), while 639 (71.4%), 633 (70.7%), 632 (70.6%) and 616 (68.8%) samples were found to be positive for brucellosis with CFT, RBT, SAT and cELISA, respectively (Table 1).

**Table 1:** Number of positive results per test used for the detection of brucellosis in asymptomatic camels

Sample n = 895	RTPCR	RBT	cELISA	CFT	SAT	FPA
534	534	534	534	534	534	534

118	118	-	-	-	-	-

57	57	-	-	-	-	57

15	15	15	-	15	15	15

5	5	-	-	5	-	5

4	4	4	4	4	-	4

4	4	4	4	4	4	-

3	3	-	3	3	3	3

3	3	3	3	-	3	-

3	3	3	-	-	3	-

2	2	2	2		2	2

2	2	-	-	-	2	2

2	2	2	-	2	2	-

1	1	1	-	-	-	1

1	1	1	-	1	-	1

1	1	-	-	1	1	1

1	1	-	1	1		1

1	1	1	-	1	-	-

1	1	1	1	-	-	-

1	1	-	-	-	1	-

61	-	61	61	61	61	61

15	-	-	-	-	-	15

4	-	-	-	4	-	4

2	-	-	2	2	-	2

1	-	1	-	-	-	1

1	-	-	1	1	1	1

52	-	-	-	-	-	-

Total n = 895	759	633	616	639	632	710

Percent %	84.8	70.7	68.8	71.4	70.6	79.3

Real-time PCR BCSP31 (Real Time Polymerase Chain Reaction, *Brucella *Cell Surface Protein 31 kDa, RBT (Rose Begnal Test), SAT(Slow Agglutination Test), CFT (Complement Fixation Test), cELISA (Competitive Enzyme Linked Immunosorbant Assay), FPA (Fluorescence Polarization Assay).

Out of 895 examined sera, 595 (66.5%) were positive and 170 (19.0%) were negative by all serological tests. 72 (8.04%) were found to be positive by FPA only. 15 samples showed false negative results by cELISA. The real-time PCR assay amplified the *Brucella *cell surface salt extractable genus specific *bcsp31 *kDa protein gene in 84.8% (759/895) samples. *B. abortus *was the only species found using species specific real-time PCR assays published by Probert et al. (2004). 534 out of 895 (59.7%) were positive by all serological tests and *bcsp31 *real-time PCR. 118 (13.2%) were positive by *bcsp31 *real-time PCR but negative in all serological tests. We observed no obvious correlation between the CT values reflecting DNA concentration with the results obtained in serological assays (data not shown). 61 (6.8%) samples were positive by serological tests but negative by *bcsp31 *real-time PCR (Table 1). The agreement between the results obtained by FPA and that for *bcsp31 *real-time PCR is illustrated in (Table 2). FPA was positive in 626 samples out of 759 (82.5%) that were positive by *bcsp31 *real-time PCR. A probit analysis revealed that real-time PCR assay detect as little as 23 fg (corresponding approximately 7 GE) of *Brucella *DNA per reaction with a probability of 95%.

**Table 2:** Comparative analysis for the results of serological test and real-time PCR

Sample		RBT		cELISA		CFT		SAT		FPA	
	
		Pos	Neg	Pos	Neg	Pos	Neg	Pos	Neg	Pos	Neg
**Bcsp31**	**Pos **(n = 759)	571	188	552	207	571	188	570	189	626	133
	
	**Neg** (n = 136)	62	74	64	72	68	68	62	74	84	52

**RBT**	**Pos** (n = 633)			609	24	622	11	624	9	619	14
	
	**Neg** **(n = 262)**			7	255	17	245	8	254	91	171

**cELISA**	**Pos** **(n = 616)**					610	6	608	8	608	8
	
	**Neg** **(n = 279)**					29	250	24	255	102	177

**CFT**	**Pos** **(n = 639)**							621	18	632	7
	
	**Neg** **(n = 256)**							11	245	78	178

**SAT**	**Pos** **(n = 632)**									619	13
	
	**Neg** **(n = 263)**									91	172

The presence of *Brucella *DNA as demonstrated by *bcsp31 *real-time PCR or presence of anti *Brucella *antibodies proved by two different serological tests was considered as proof for a potential risk of consumers when consuming products of these animals. The panel of sera which fulfill at least one of these criteria was considered to be the "gold standard". According to this definition (positive by real-time PCR or two different serological tests) 828 samples had to be considered as "true" positive. Real-time PCR detects 759 samples out of 828 with a sensitivity of 91.7%. The sensitivity of RBT, cELISA, CFT, SAT and FPA was 76.5, 74.4, 77.2, 76.3 and 83.9%, respectively. The LCA model with two classes showed a good fit (BIC = 2931.761) for two latent classes, which demonstrates that the underlying correlation structure can be well explained by two latent classes.

Real-time PCR had a high sensitivity but low specificity in relation to the latent class, whereas the serological tests all showed very high sensitivity and specificity (Table 3). The Chi-Square test showed a highly significant correlation of all tests with the latent class (p < 10^12 ^or lower).

**Table 3:** Correlation of tests calculated according to the latent class model

Test	Correlation [%]		
	
	Total	Positive	Negative
Real-time PCR	72	90	28

RBT	99	99	97

cELISA	97	97	99

CFT	98	99	95

SAT	99	99	98

FPA	89	99	67

## Discussion

Control of brucellosis in livestock and humans depends on the reliability of the methods used for detection and identification of the causative agent. However, diagnosis of brucellosis in camels is frequently difficult. The disease can mimic many infectious and non infectious diseases. Characteristic clinical signs of brucellosis in camels are often lacking and diagnostic methods are not evaluated yet. In the present study, all camels were clinically normal at the time of sampling and according to the owners, none had previously shown clinical signs of brucellosis. Theses results indicate that many infected camels might be silent carriers for brucellosis and their products may pose a serious health problem for consumers. Our observations are supported by a study [15] demonstrating that non pregnant camels experimentally infected with a field strain of *B. abortus *had no clinical signs and only negligible pathological changes were present. The authors found also that the organism was recovered mainly from the lymph nodes of the head and genital tract. However, single cases of abortion, placental retention, fetal death, mummification, delayed sexual maturity, infertility, stillbirth, mastitis, orchitis and joint disease have been reported from camels naturally infected with *B. abortus *[16]. Having in mind these facts, a camel posing a risk for consumers was considered either to have *Brucella *DNA in its blood samples or being positive for the presence of antibodies confirmed by two independent serological test systems. Animals that are positive for DNA only may be in the incubation period before an antibody titer develops or may simply be unable to produce specific antibodies at all.

Serological tests can detect infection when sepsis has passed and the agent has found its niche in the host. The obtained results revealed that 79.3, 71.4, 70.7, 70.6 and 68.8% of the camel sera investigated were positive by FPA, CFT, RBT, SAT and cELISA, respectively. CFT detected more positive cases than agglutination tests. This result is in agreement with that previously reported [17,18]. These authors stated that the complement fixation test is the most widely used test for brucellosis screening in camels. Only little information was available in the literature on the application of cELISA on camel serum [18,19]. Various studies, however, have confirmed that ELISA techniques perform better than other conventional tests used for serological screening of brucellosis in other animal species [20]. Our results revealed that 616 camel serum samples out of 828 (74.4%) were positive by cELISA, demonstrating the lowest sensitivity when compared to other serological tests. This may be attributed to the fact that cELISA was specially standardized to work with bovine sera or the very special presentation of brucellosis in camels. It has to be stressed that sensitivity and specificity may vary considerably if another cELISA will be tested. To the best of our knowledge, FPA has not been used for the diagnosis of camel brucellosis yet. Our results revealed that FPA detected more positive cases than all other serological tests used. Thus, FPA seems to be a valuable tool for the diagnosis of brucellosis in camels especially when taking into consideration the speed, the objectivity of result interpretation and the cost factor. FPA could be considered as a senseful replacement for other established methods. Further studies are now needed to assess FPA's reproducibility. A perfect agreement between CFT, RBT and SAT was proven by calculating Kappa values but sensitivity of all tests is low when compared to FPA or real-time PCR. It is advisable to combine at least two serological test methods to screen brucellosis on herd level. This finding is in accordance with the procedure of monitoring in other animal species. Thus, the sensitivity will be increased. Nevertheless, serological methods used solitary or in combination carry the risk to miss seronegative carriers of *Brucella*.

Real-time PCR proved to be a valuable diagnostic tool when culture fails or serological results are inconclusive in human brucellosis [20]. It is faster and more sensitive than culture. The risk of transmission of brucellosis to laboratory workers can be minimized. To the best of our knowledge real-time PCR has not been previously used for diagnosis of camel brucellosis. Interestingly, real-time PCR targeting the genus specific *bcsp31 *was positive in 84.8% out of 895 samples demonstrating the presence of the agent within the animal. It cannot be concluded that these camels are permanently infected, although brucellosis tends to have a chronic course. However, these animals may be asymptomatic carriers and shedders. They pose a permanent risk to other animals and humans and have to be removed from the herd.

534 samples out of 895 (59.7%) were positive in all serological tests and real-time PCR. The high percentage of positive animals detected by real-time PCR could be attributed to the high diagnostic sensitivity of the real-time PCR assay to detect as little as 23 fg of *Brucella *DNA per reaction with a probability of 95%. Real-time PCR detected also infection in 118 seronegative camels which prompts us to conclude that these were probably acute or chronically infected animals with antibody levels not yet or no longer detectable. While PCR directly detects the DNA of the pathogen, serology is dependent upon the rising and falling titers of antibodies during the different phases of brucellosis. Consequently, real-time PCR is the test to complement diagnosis of camel brucellosis. We suggest that conventional PCR could be used in developing countries because of the lower costs and the high amount of *Brucella *DNA in the blood of infected camels in contrast to other animal species. 15.2% (136/895) of samples were negative by real-time PCR. These samples include 61 which were seropositive in our study. In fact, this subpopulation is smaller than expected, because this constellation would indicate a passed infection without circulating DNA but antibodies present in serum. On the herd level a high number of chronic infections would have a negative impact on the usability of real-time PCR as a screening tool, but it may have advantages in acute cases when there is bacteremia but no immunological response or clinical picture yet. The storage time can negatively influence the presence of detectable DNA as well as the antibody concentration. A storage time of up to one year as occured in our study might have some influence on the performance of the assays.

Assuming that the two latent classes truly reflect the infection status of the animals the LCA results indicate that the real-time PCR has the worst performance. The serological tests all perform concordant and very well. However, the LCA cannot answer the question, whether the real-time PCR provides false positive results or whether in certain cases all other tests provide the wrong result. As the LCA per se has no preference for a single method, the serological results may dominate and will bias the overall result. In our view the biological interpretation is that in early infection all serological tests are still negative and the PCR assays are true positive while in passed infections the pathogen DNA has been eliminated by the organism. So in acute infections real-time PCR is a relevant and useful method while in chronic and passed infection seroconversion is significantly more reliable. With regard to consumer protection the LCA analysis does not help to estimate the value of a single diagnostic test. In our interpretation the presence of *Brucella *DNA justifies the removal of animals according to OIE guidelines.

The high percentage of positive cases might be caused by the fact that the camels were imported from Sudan where a high prevalence of camel brucellosis is known [21-23]. Nevertheless, seroprevalence of brucellosis in camels has to be examined in confirmatory studies to evaluate the importance of the disease in camel population. Isolation of *B. abortus *from free ranging camelids in Sudan was already reported [2,17].

In accordance a species specific real-time PCR system revealed that only *B. abortus *was present in the serum of the camels investigated in this study. It can be supposed that a spill over from cattle was the origin for this findings and the spread of disease was promoted by the crowding situation during transport. This fact does not ensure that these camels are infected with *B. abortus *only since *B. melitensis *was isolated from camels of the same origin earlier (data not shown).

## Conclusion

In developed countries, a combination of real-time PCR with at least one serological method preferably FPA could be applied to detect brucellosis in camels. In less developed countries a combination of conventional PCR with one of the commonly used serological tests (i.e. RBT, SAT, CFT) can be recommended. Camels have to be included in national programs for control and eradication of brucellosis in endemic countries.

## Competing interests

The authors declare that they have no competing interests.

## Authors' contributions

MMG designed and coordinated the study, carried out the experimental work and drafted the manuscript. AHE, UR, MCE, RW, UW and IK helped to draft the manuscript. FM participated in study design and helped to draft the manuscript. HN and HT participated in the design of the study, the evaluation of the PCR assays and helped to draft the manuscript. DS and EM did the statistical analysis of the data. All authors read and approved the final manuscript.
